# Efficacy and Safety of Scalp Acupuncture for Insomnia: A Systematic Review and Meta-Analysis

**DOI:** 10.1155/2021/6621993

**Published:** 2021-05-22

**Authors:** Fu-gui Liu, Ai-hua Tan, Chao-qun Peng, Yun-xia Tan, Ming-chao Yao

**Affiliations:** ^1^Clinical College of Traditional Chinese Medicine, Hubei University of Chinese Medicine, Wuhan 430065, Hubei, China; ^2^Institute of Geriatrics, Hubei University of Chinese Medicine, Key Laboratory of Alzheimer's Disease (XINGNAOYIZHI), State Administration of Traditional Chinese Medicine, Wuhan 430065, China; ^3^The First Clinical Medical College of Henan University of Chinese Medicine, Zhengzhou 450046, Henan, China; ^4^College of Rehabilitation Medicine, Henan University of Traditional Chinese Medicine, Zhengzhou 450046, Henan, China

## Abstract

**Objective:**

To systematically evaluate the efficacy and safety of scalp acupuncture in the treatment of insomnia.

**Methods:**

CNKI, Wanfang database, CQVIP database, CBM, Web of Science, Cochrane Library, and PubMed were searched for the literature on the treatment of insomnia by scalp acupuncture from the establishment of the database to July 23, 2020. Two researchers independently screened the literatures and extracted the data, then evaluated the quality of the literatures, and used RevMan 5.3 software for statistical analysis.

**Results:**

A total of 21 studies including 1606 cases were included. 21 studies were included in the analysis of effective rate. The heterogeneity test showed that there was no significant heterogeneity. The fixed effect model was used, *P* < 0.00001. The effective rate of scalp acupuncture in the treatment of insomnia was significantly higher than that of the control group. The analysis of PSQI score was finally included in 19 studies. The heterogeneity test showed that there was obvious heterogeneity. The random effect model was used, and the subgroup analysis was conducted according to the different intervention measures of the control group. The *P* values of the drug group and the blank group were both less than 0.05, indicating that the improvement of PSQI score in the scalp acupuncture treatment of insomnia was significantly better than that in the drug group and the blank group; *P* = 0.05 in other acupuncture groups, suggesting in scalp acupuncture treatment, there was no difference between insomnia and other acupuncture in improving the PSQI score. Six studies were included in the analysis of adverse events. The heterogeneity test showed no significant heterogeneity. The fixed effect model was used, *P* = 0.04 < 0.05, indicating that the adverse events of scalp acupuncture in the treatment of insomnia were better than those of the control group. No publication bias analysis was conducted due to the small number of adverse events included. Publication bias was analyzed for effective rate and PSQI score. Egger's TSTs test (effective rate *P* = 0.001, PSQI score *P* = 0.001) and funnel plot showed publication bias.

**Conclusion:**

Scalp acupuncture is effective and safe in the treatment of insomnia, which is worthy of clinical application. However, due to the limited number of included literature, the methodology of some studies is slightly low and the quality of literature is slightly poor. In the future, we need to design rigorous, large sample, multiple center randomized controlled study to further verify the conclusion of this study.

## 1. Introduction

Insomnia refers to a subjective experience of dissatisfaction with sleep time and (or) quality under the appropriate sleep opportunity and sleep environment, which affects the social function during the day [1]. With the development of social economy, science, and technology, and the acceleration of life rhythm, people's pressure is increasing. Insomnia has become the most common sleep disorder disease in the population, and it is also the most common disease in sleep disorder clinic. It has been reported that [1–4] the average insomnia rate in the world is 27%, as high as 31.2% in China, and the persistent rate of insomnia in adults is 30%–60%. About 50% of patients will develop into chronic insomnia. The etiology and pathophysiology of insomnia involve heredity, physiology, environment, behavior, gender, spirit and emotion, etc. Long-term insomnia not only affects the daily life, study, and work of patients, but also increases the risk of suffering from other diseases, causing greater psychological and economic burden to patients and their families [5, 6]. At present, drugs are the main treatment, but the side effects and dependent effects of drugs make people constantly explore better treatment methods [7]. Acupuncture, as one of the traditional Chinese medical therapies, can alleviate or cure diseases by stimulating specific acupoints with acupuncture needles. It has been recognized by more and more people all over the world because of its advantages such as being easy to carry, quick effect, almost no side effect, and no dependence [3]. As one of the acupuncture therapies, scalp acupuncture is also called head acupuncture. It stimulates some acupoints in the head through the application of acupuncture, so as to achieve the purpose of prevention and treatment of systemic diseases. At present, it has been widely used in clinical practice. However, the efficacy and safety of scalp acupuncture in the treatment of insomnia has not been unified, and no one has conducted a systematic evaluation on it. This study aims to systematically evaluate the efficacy and safety of scalp acupuncture in the treatment of insomnia, so as to provide reference for clinical practice.

## 2. Data and Methods

### 2.1. Retrieval Strategy

This review was executed according to the guidance in the “Preferred Reporting Items for Systematic Reviews and Meta-Analyses” (PRISMA) statement. The protocol was prospectively registered in the International Prospective Register of Systematic Reviews (PROSPERO) database in Oct 11, 2020 (registration number:CRD42020203340).

With “insomnia,” “agrypnia,” “hyposomnia,” “sleeplessness,” “dyssomnia,” “sleep disorder,” “scalp acupuncture,” “head acupuncture,” “scalp-acupuncture,” “random,” “stochasticas,” and “randomly” as the subject words and free words, we searched CNKI (China National Knowledge Infrastructure, https://www.cnki.net/), CQVIP (WEIPU information network, https://www.cqvip.com.), Biology Medicine disc, http://www.sinomed.ac.cn DATA (Wanfang database, http://www.wanfangdata.com), Web of Science (http://apps.webofknowledge.com.) Library (https://www.cochranelibrary.com/), and PubMed (https://pubmed.ncbi.nlm.nih.gov/) from the establishment of the database to July 23, 2020. The specific retrieval strategy takes PubMed as an example, as given in Table 1:

### 2.2. Inclusion and Exclusion Criteria

#### 2.2.1. Inclusion Criteria

(1) The study was a randomized controlled trial, regardless of whether it was a blind method or not, and the language was not limited; (2) the subjects were patients with insomnia who were definitely diagnosed, regardless of gender, age, race, color of skin, and nationality. The diagnostic criteria referred to the diagnostic criteria of chronic insomnia in the international classification of sleep disorders (3rd Edition) [5, 6]; (3) the intervention measures in the treatment group were scalp acupuncture or head acupuncture alone for insomnia; (4) there was a clear treatment course; (5) the observation indexes were effective rate, PSQI score, and adverse events.

#### 2.2.2. Exclusion Criteria

They were as follows: (1) nonrandomized controlled trials; (2) case reports, clinical experience, research progress, animal experiments; (3) the intervention measures of the control group being scalp acupuncture alone or combined therapy; (4) the literatures published repeatedly or published by more than one person in the same study being excluded until one of the published articles was the latest and the most complete data was retained; (5) serious lack of data affecting the study, or attempting to contact the author not yet able to obtain the literature with missing data.

### 2.3. Data Extraction

Two researchers independently completed the screening according to the inclusion and exclusion criteria. NoteExpress software was used to manage the literature. Repetitive and nonconforming literatures were excluded by title, abstract, and full-text reading, then Excel form was used to extract data independently from the included literature, and the included literature and extracted data were cross-checked. The contents of data extraction included the author, year of publication, intervention measures of the experimental group and the control group, the number of cases in the experimental group, the number of cases in the control group, the number of male cases, the number of female cases, the age of inclusion, the course of treatment, the random method, the number and causes of shedding cases, observation indicators, and adverse reactions. The third researcher discussed and decided on the content of the literature and materials with objection.

### 2.4. Literature Quality Assessment

Two researchers independently evaluated the quality of the included literature according to Cochrane system evaluation manual [8]. The evaluation contents included random sequence generation, allocation concealment, observer and testee blinding, result evaluation blinding, result data integrity, selective reporting, and other biases.

### 2.5. Statistical Analysis

RevMan 5.3 software was used for statistical analysis of the data. Odds ratio (or) was used as the statistical value for categorical variable data, and weighted mean difference (WMD) or standardized mean difference (SMD) was used for continuous variable data. Each effect size was represented by 95% CI, *P* < 0.05, and the difference was considered statistically significant. Q test and I^2^ test were used for heterogeneity analysis, *P* > 0.1, I^2^ < 50%, considered no or mild heterogeneity, and fixed effect model was used; *P* < 0.1, I^2^ > 50%, considered that there was heterogeneity, random effect model was used, and subgroup analysis was conducted to find the possible causes of heterogeneity. By excluding the included literatures one by one, the sensitivity analysis of the outcome indicators with high heterogeneity was carried out. Through the transformation effect model, the sensitivity analysis of the outcome indicators with small heterogeneity was carried out to evaluate the impact of the included studies on the robustness of the final results. If the heterogeneity of subgroup analysis and sensitivity classification is still high, meta-regression is used to further analyze the sources of heterogeneity. Funnel plot was used to analyze publication bias, and Stata 14 software was used to conduct Egger's test to quantitatively evaluate publication bias.

## 3. Results

### 3.1. Literature Search Results

**Figure 1:**
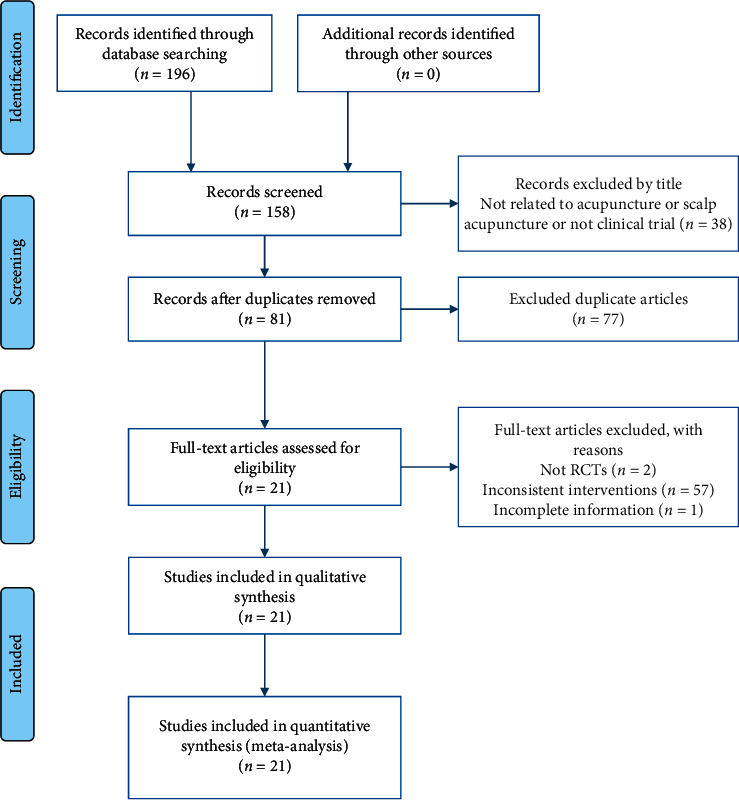
Flow chart of literature screening.

A total of 196 articles were initially searched, and the inconsistent literatures were excluded through the title, and then repetitive literatures were excluded, except for the ones with the latest publication time and the most complete data. Through the summary, and reading the full text to complete the screening, 21 literatures were finally included. The specific literature screening process is shown in Figure 1.

### 3.2. Basic Information of Included Literatures

A total of 21 articles were included, including 1 in English and 20 in Chinese. A total of 1606 cases were included, including 804 cases in the treatment group and 802 cases in the control group. The intervention measures in the treatment group included scalp acupuncture, head acupuncture, scalp point penetration needling, scalp cluster needling, and head through point needling. When extracting information, the above expressions were uniformly named as scalp acupuncture, while the intervention measures in the control group included body acupuncture, conventional acupuncture, general acupuncture, right left picrolon, eszolam, oryzanol, clozapine, and clonazepam. The course of treatment involved in the included literature included 7 days, 10 days, 14 days, 20 days, 21 days, 28 days, 30 days, and 40 days. The basic information of the included literature is shown in Table 2.

### 3.3. Included Literature Quality Evaluation

**Figure 2:**
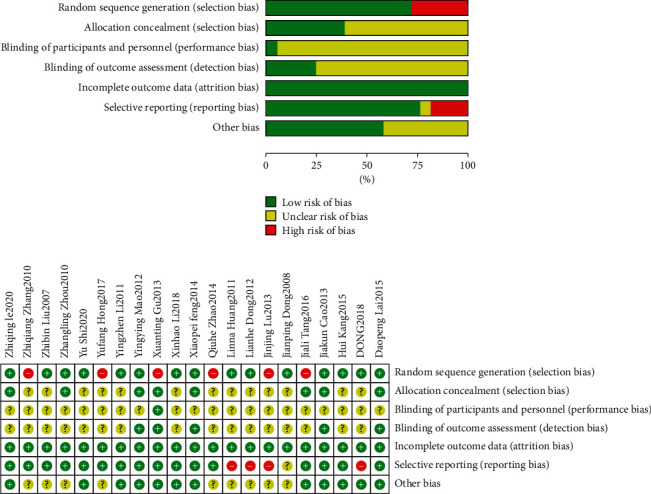
Literature quality risk bias chart.

The included literatures were randomized into groups, including the following: 12 articles [9–20] were randomized into groups by random number table method, 4 articles [17, 21–23] were randomly divided into groups by statistical software, 1 article [24] was randomly divided according to the order of treatment, and 4 articles [25–28] did not mention the specific random grouping method. 6 studies [16–18, 21, 23, 27] used allocation concealment, and 15 [9–15, 19, 20, 22, 24–26, 28, 29] did not mention allocation concealment. 1 [18] study used single blind method, 20 [9–17, 19–29] studies did not mention blind method. Five studies [16–18, 21, 23] blinded the result evaluation, and 16 studies [9–15, 19, 20, 22, 24–29] did not mention whether to blind the result evaluation. Four studies [9, 12, 15, 28] had reporting bias, and 17 [10, 11, 13, 14, 16–27, 29] had no reporting bias. There were no other biases in 12 studies [9, 13, 14, 16–23, 27] and 9 [10–12, 15, 24–26, 28, 29] studies were not clear on other bias. The specific literature quality evaluation is shown in Figure 2.

### 3.4. Analysis of the Effective Rate

**Figure 3:**
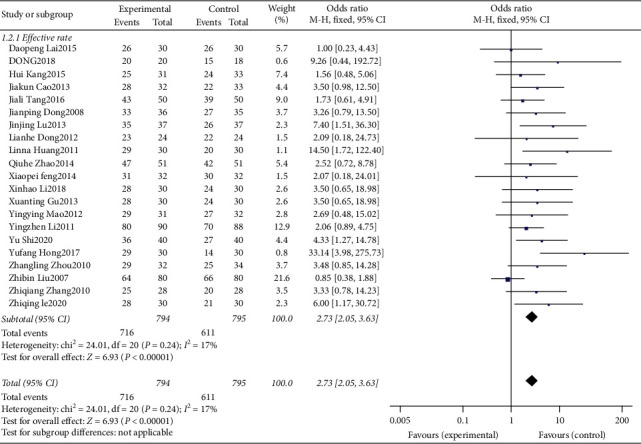
The forest plot of effective rate.

The outcome indicators of all studies included effective rate; finally, 21 studies were included. The heterogeneity test result is p = 0.24, I2 = 17%, which shows that there is no heterogeneity, so the fixed effects model is selected. The combined effect amount results showed that OR = 2.73, 95% CI [2.05, 3.63], *P* < 0.00001 (Figure 3), the difference was statistically significant, and the effective rate of scalp acupuncture in the treatment of insomnia was significantly higher than that in the control group. Because the heterogeneity test showed no significant heterogeneity, subgroup analysis was not performed.

### 3.5. Analysis of PSQI Score

**Figure 4:**
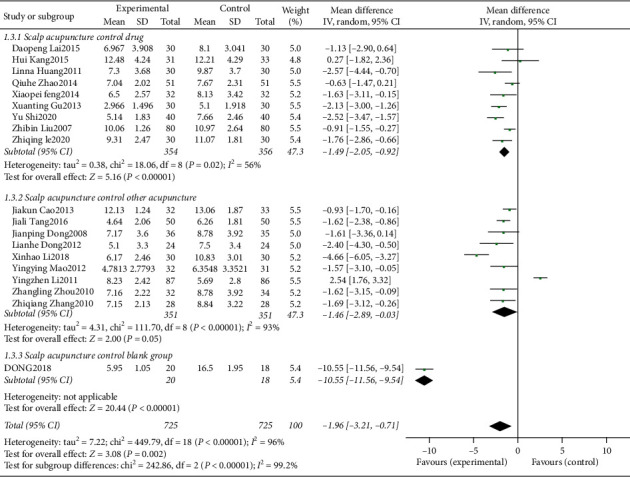
The forest plot of PSQI score.

PSQI is the Pittsburgh Sleep Quality Index (PSQI), which is a self-report questionnaire for measuring sleep quality. It includes seven dimensions: fall asleep time, sleep quality, sleep time, sleep efficiency, hypnotic drugs, sleep disorders, and daytime dysfunction. The lower the score, the higher the sleep quality [30]. There are 2 [24, 28] study outcome indicators without PSQI, and 19 [9–23, 25–27, 29] studies were finally included, of which 9 [10–12, 14, 21–23, 25, 27] studies used other acupuncture as the control intervention measures, 9 studies [10–12, 14, 21–23, 25, 27] used drugs as the control group interventions, and 1 [9] study's control group intervention was blank treatments. The heterogeneity test showed significant heterogeneity (*P* < 0.00001, *I*^2^ = 96%), using random effect model. The results of combined effect amount showed that MD = −1.96, 95% CI [−3.21, −0.71], *P* = 0.002; the difference was statistically significant, which means that scalp acupuncture for insomnia improves the PSQI score better than the control group. According to the different intervention measures in the control group, 19 studies were divided into scalp acupuncture control drug group, scalp acupuncture control other acupuncture groups, and scalp acupuncture control blank group. Subgroup analysis results (Figure 4) showed that the improvement of PSQI score of insomnia patients by scalp acupuncture was significantly better than that of drug group (MD = −1.49, 95% CI [−2.05, −0.92], *P* < 0.00001, heterogeneity test: *P* = 0.02, *I*^2^ = 56%) and blank group (MD = −10.55, 95% CI [−11.56, −9.54], *P* < 0.00001, because there was only one study; there was no heterogeneity test), compared with other acupuncture groups (MD = −1.46, 95% CI [−2.89, −0.03], *P* = 0.05, heterogeneity test: *P* < 0.00001, *I*^2^ = 93%); there was no significant difference. Subgroup analysis showed that both drug group and acupuncture group had heterogeneity; considering the heterogeneity of PSQI score, it was related to different intervention measures in control group.

### 3.6. Analysis of Adverse Events

**Figure 5:**
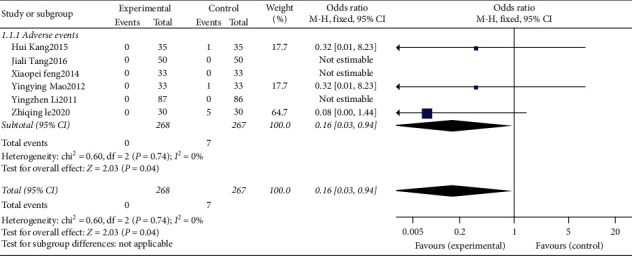
The forest plot of adverse events.

There was 1 study [21] with dizziness in the control group, 1 [20] with nausea in the control group, 1 [13] with dry mouth and abnormal taste in the control group, 3 [17, 22, 27] studies that did not find any adverse reactions, and 15 studies [9–12, 14, 15, 18, 21, 23–29] that did not mention adverse reactions. Finally, 6 studies [9–12, 14, 15, 18, 21, 23–29] were included, with a total of 535 cases, 268 cases in the treatment group and 267 cases in the control group. The heterogeneity test showed that *P* = 0.74, I2 = 0%, no obvious heterogeneity, and a fixed effects model was used. The combined effect size results showed that OR = 0.16, 95%CI [0.03, 0.94], *P* = 0.04 (Figure 5). The difference was statistically significant. The scalp acupuncture treatment of insomnia was significantly better than the control group in the occurrence of adverse events. Since the heterogeneity test showed no obvious heterogeneity, the subgroup analysis was no longer performed.

## 4. Sensitivity Analysis

Outcome indicators that have no obvious heterogeneity in the heterogeneity test, such as effective rate and adverse events, are subjected to sensitivity analysis using the conversion effect model. Outcome indicators that have obvious heterogeneity in the heterogeneity test, such as PSQI score, are eliminated by one-by-one method to conduct sensitivity analysis.

**Figure 6:**
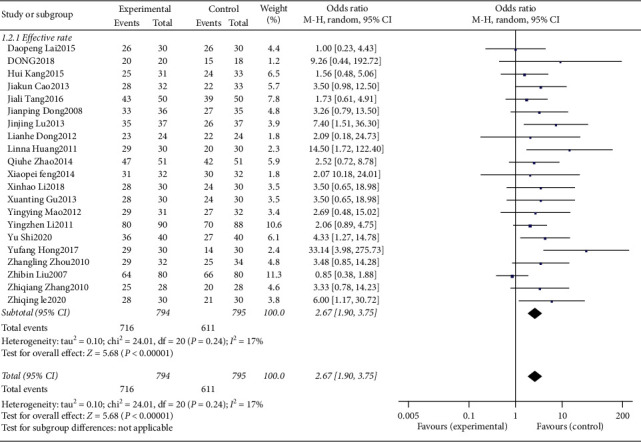
Sensitivity analysis of effective rate.

**Figure 7:**
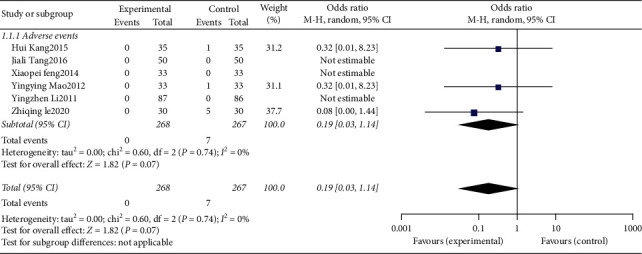
Sensitivity analysis of adverse events.

**Figure 8:**
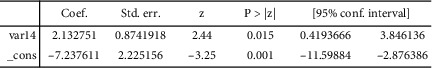
Meta-regression analysis of PSQI score.

Sensitivity analysis of effective rate (Figure 6) showed that OR = 2.67, 95%CI [1.90, 3.75], *P* < 0.00001, the effective rate of scalp acupuncture in the treatment of insomnia is still better than the control group, and the effective rate meta-analysis results are robust. The sensitivity analysis of adverse events (Figure 7) shows that OR = 0.31, 95%CI [0.07, 1.40], *P* = 0.13; the adverse reactions of scalp acupuncture treatment of insomnia are still not significantly different from those of the control group. The results of meta-analysis of adverse reactions are steady. The sensitivity analysis of PSQI score (Table 3) showed that the *P* values after excluding each study were all < 0.05, and the difference was statistically significant. The improvement of PSQI score by scalp acupuncture in the treatment of insomnia was still better than that of the control group, and the meta-analysis results were robust. However, there was a high heterogeneity when excluding any study. Therefore, according to the different interventions of the control group, the PSQI score was analyzed by meta-regression analysis to further analyze the source of heterogeneity. Meta-regression analysis showed that *P* = 0.015 < 0.05 (see Figure 8), which was statistically significant. Therefore, different interventions in the control group could explain the source of heterogeneity.

## 5. Publication Bias Analysis

**Figure 9:**
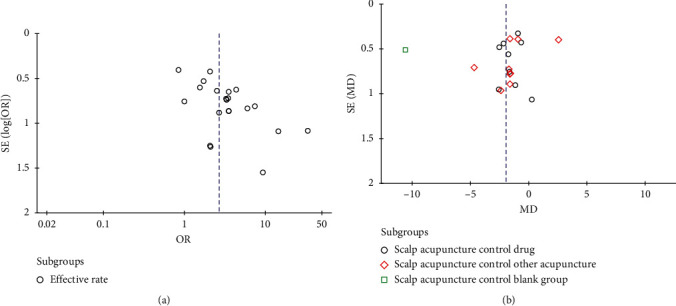
(a) The funnel plot of effective rate; (b) the funnel plot of PSQI score.

**Figure 10:**
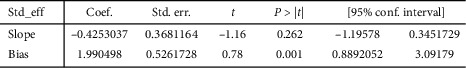
Egger's test results of effective rate.

**Figure 11:**
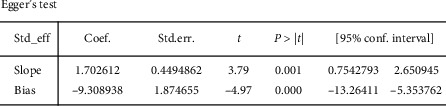
Egger's test results of PSQI score.

Due to the small number of adverse events included in the study, funnel plot analysis was no longer performed. The funnel plot of effective rate (Figure 9(a)) and PSQI score (Figure 9(b)) shows the presence of publication bias. The effective rate Egger's test (Figure 10) results show that *P* = 0.001, and the Egger's test results of PSQI score (Figure 11) show *P* = 0.001, which are consistent with funnel plot results, indicating that there was publication bias, which may be related to the failure to retrieve gray literature and the journals and researchers tend to publish studies with positive results.

## 6. Discussion

Acupuncture which is a medical therapy that has been proved to have obvious curative effect on various diseases by clinical practice plays a role in regulating qi and blood of viscera, meridians, and collaterals by acting on different meridians and acupoints [31]. According to the characteristics of diseases, different acupuncture forms such as scalp acupuncture, warm acupuncture, abdominal acupuncture, and body acupuncture can be selected in clinic. Although scalp acupuncture is a kind of acupuncture, it is also a special external treatment of traditional Chinese medicine different from ordinary acupuncture. First of all, scalp acupuncture is a specific part of the acupuncture head, because the head has a skull, so the safety of scalp acupuncture is higher than ordinary acupuncture; secondly, because of the particularity of scalp acupuncture, it not only has the effect of dredging meridians and collaterals by ordinary acupuncture, but also has the characteristics of targeted stimulation of cerebral cortex function areas that ordinary acupuncture does not have, so it has more advantages in the treatment of neurological diseases [32]. According to the basic theory of traditional Chinese medicine that “the head is the capital of Qingyang,” “the meeting of all yang,” “all meridians are attributed to the brain,” and “the essence and qi of five-internal six organs all rise above the head,” acupuncture on the head has a stronger regulating effect on the qi movement of the whole body [33]. Head acupuncture is more friendly to headache, insomnia, and other neurological diseases because of its unique role, with the rising number of insomnia cases, the side effects of sleeping pills are obvious, so scholars have a strong interest in acupuncture treatment of insomnia. Current studies have found that scalp acupuncture can not only treat primary insomnia, but also treat insomnia caused by other diseases, such as perimenopausal insomnia and Parkinson's insomnia [15, 34, 35]. At present, the mechanism of scalp acupuncture in the treatment of insomnia is still not conclusive, and modern basic research has carried out a lot of exploration. Some studies have found that [18] scalp acupuncture can increase the concentration of cytokines IL-1 *β*, TNF-*α*, and IL-6 in the brain tissue of insomnia rats; some studies have found that [16] scalp acupuncture may affect the secretion of neurotransmitters and regulate the central organs that affect sleep wakefulness; some studies [36] have found that using scalp acupuncture to stimulate insomnia's areas of interest to varying degrees can promote the balance of physiological functions of the cerebral cortex and regulate the autonomic nervous system. With people's awareness of the side effects of drugs in the treatment of insomnia, there are more and more clinical studies on the treatment of insomnia with scalp acupuncture alone or in combination. The systematic evaluation of the efficacy and safety of scalp acupuncture in the treatment of insomnia has great clinical significance.

Through literature search, it is found that although there is a meta-analysis on the efficacy and safety of scalp acupuncture in the treatment of insomnia, the control group is mostly other forms of acupuncture or sham acupuncture [37, 38]. The control group of this study is not limited to other forms of acupuncture, but also includes commonly used sleeping pills in clinic. In addition, this paper not only uses the Pittsburgh sleep quality questionnaire score as the outcome evaluation index, but also uses the efficiency and adverse reactions as the outcome evaluation index, which increased the credibility of the results of this study to a certain extent. A total of 21 studies were included in this study, and a total of 1606 cases were included. The comprehensive evaluation results show that scalp acupuncture is effective in treating insomnia. Compared with the control group, scalp acupuncture can significantly improve the effective rate of treating insomnia. The PSQI score subgroup analysis showed that the PSQI score of the head was significantly better than that of the drug and blank group, but there was no significant difference from other acupuncture methods. Sensitivity analysis showed that scalp acupuncture ameliorates the PSQI score better, but excluding any item had high heterogeneity. Meta regression analysis of PSQI score according to different intervention measures in the control group showed that the difference of intervention measures in the control group was the source of heterogeneity. In terms of adverse events, the scalp acupuncture group had no adverse events, which was significantly better than the control group. Based on this study, we found that scalp acupuncture is effective and safe in the treatment of insomnia.

This study has some limitations. Due to the small number of included literatures and the limited sources of literatures, the quality of some studies is slightly poor. In the future, a large number of rigorously designed, large-sample, multi-center randomized controlled studies are needed to further verify the results of this study.

## 7. Conclusion

According to the results of this study, scalp acupuncture is effective in the treatment of insomnia, and its efficacy and safety are better than those of drugs and blank treatments. It has the value of clinical promotion and application, but scalp acupuncture has no significant difference in efficacy and safety compared with other acupuncture treatments for insomnia. The quality of the included literature, the source of the literature, the design of the literature research, and the intervention measures of the control group may have affected the final result to some extent.

## Figures and Tables

**Table 1:** Search strategy.

#1 insomnia [Title/abstract]
#2 agrypnia [Title/abstract]
#3 hyposomnia [Title/abstract]
#4 sleeplessness [Title/abstract]
#5 sleep disorder [Title/abstract]
#6 dyssomnia [Title/abstract]
#7 #1 OR #2 OR #3 OR #4 OR #5 OR #6
#8 insomnia [MeSH terms]
#9 agrypnia [MeSH terms]
#10 hypersomnia [MeSH terms]
#11 sleeplessness [MeSH terms]
#12 sleep disorder [MeSH terms]
#13 dyssomnia [MeSH terms]
#14 #8 OR #9 OR #10 OR #11 OR #12 OR #13
#15 #7 OR #14
#16 scalp acupuncture [Title/abstract]
#17 head acupuncture [Title/abstract]
#18 scalp-acupuncture [Title/abstract]
#19 #16 OR #17 OR #18
#20 scalp acupuncture [MeSH terms]
#21 head acupuncture [MeSH terms]
#22 scalp-acupuncture [MeSH terms]
#23 #20 OR #21 OR #22
#24 #19 OR #23
#25 random [MeSH terms]
#26 stochastic [MeSH terms]
#27 randomly [MeSH terms]
#28 #25 OR #26 OR #27
#29 random [Title/abstract]
#30 randomly [Title/abstract]
#31 stochastic [Title/abstract]
#32 #29 OR #30 OR #31
#33 #28 OR #32
#34 #15 AND #24 AND #33

**Table 2:** Basic information of the included literatures.

First author	Year of publication	Languages	Interventions		Number of cases		Course of treatment	Outcome indicators
			Treatment group	Control group	Treatment group	Control group		
Dong	2018	English	Scalp acupuncture	No treatment	20	20	28 days	①②
Jianpian Dong	2008	Chinese	Scalp acupuncture	Routine acupuncture	36	35	30 days	①②
Zhangling Zhou	2010	Chinese	Scalp acupuncture	Routine acupuncture	35	35	28 days	①②
Yingzhen Li	2011	Chinese	Scalp acupuncture	Body acupuncture	87	86	28 days	①②③
Lianhe Dong	2012	Chinese	Scalp acupuncture	Body acupuncture	24	24	28 days	①②
Zhiqing le	2020	Chinese	Scalp acupuncture	Right left picrolon	30	30	30 days	①②③
Zhiqiang Zhang	2010	Chinese	Scalp acupuncture	Routine acupuncture	28	28	30 days	①②
Jiakun Cao	2013	Chinese	Scalp acupuncture	Routine acupuncture	35	35	30 days	①②
Xuanting gu	2013	Chinese	Scalp acupuncture	Estazolam	30	30	14 days	①②
Qiuhe Zhao	2014	Chinese	Scalp acupuncture	Estazolam and oryzanol	51	51	20 days	①②
Xinhao Li	2018	Chinese	Scalp acupuncture	Routine acupuncture	30	30	28 days	①②
Jiali Tang	2016	Chinese	Scalp acupuncture	Routine acupuncture	50	50	30 days	①②③
Yu Shi	2020	Chinese	Scalp acupuncture	Diazepam	40	40	21 days	①②
Linna Huang	2011	Chinese	Scalp acupuncture	Estazolam	30	30	20 days	①②
Yufang Hong	2017	Chinese	Scalp acupuncture	Routine acupuncture	30	30	28 days	②
Zhibin Liu	2007	Chinese	Scalp acupuncture	Clonazepam	80	80	28 days	①②
Yingying Mao	2012	Chinese	Scalp acupuncture	Routine acupuncture	33	33	28 days	①②③
Daopeng Lai	2015	Chinese	Scalp acupuncture	Estazolam	30	30	7 days	①②
Jinchen Lu	2013	Chinese	Scalp acupuncture	Body acupuncture	37	37	40 days	②
Hui Kang	2015	Chinese	Scalp acupuncture	Estazolam	35	35	28 days	①②③
Xiaopei Feng	2014	Chinese	Scalp acupuncture	Clozapine	33	33	10 days	①②③

①PSQI score; ②effective rate; ③adverse events.

**Table 3:** Sensitivity analysis of the PSQI score.

Excluded studies	OR	95%CI	*P*	*I* ^2^ (%)	Q test *P* value
Zhiqing Le (2020)	−2.05	[−3.41, −0.69]	0.003	96	*P* < 0.00001
Xiaopei Feng (2014)	−2.06	[−3.40, −0.72]	0.003	96	*P* < 0.00001
HuiKang (2015)	−2.15	[−3.48, −0.83]	0.001	96	*P* < 0.00001
YingzhenLi (2011)	−2.01	[−3.34, −0.68]	0.003	96	*P* < 0.00001
Jiali Tang (2016)	−2.01	[−3.34, −0.68]	0.003	96	*P* < 0.00001
Yingying Mao (2012)	−2.01	[−3.34, −0.68]	0.003	96	*P* < 0.00001
Zhibin Liu (2007)	−2.1	[−3.52, −0.68]	0.004	96	*P* < 0.00001
Yu Shi (2020)	−2.01	[−3.38, −0.64]	0.004	96	*P* < 0.00001
Daopeng Lai (2015)	−2.01	[−3.34, −0.68]	0.003	96	*P* < 0.00001
Qiuhe Zhao (2014)	−2.04	[−3.32, −0.75]	0.002	96	*P* < 0.00001
Xuanting Gu (2013)	−2.04	[−3.32, −0.75]	0.002	96	*P* < 0.00001
Linna Huang (2011)	−2.01	[−3.34, −0.68]	0.003	96	*P* < 0.00001
Zhangling Zhou (2010)	−2.01	[−3.34, −0.68]	0.003	96	*P* < 0.00001
Zhiqiang Zhang (2010)	−2.04	[−3.32, −0.75]	0.002	96	*P* < 0.00001
Jiakun Cao (2013)	−2.04	[−3.32, −0.75]	0.002	96	*P* < 0.00001
Xinhao Li (2018)	−2.01	[−3.34, −0.68]	0.003	96	*P* < 0.00001
Jianping Dong (2008)	−2.01	[−3.34, −0.68]	0.003	96	*P* < 0.00001
Lianhe Dong (2012)	−2.01	[−3.34, −0.68]	0.003	96	*P* < 0.00001
Dong (2018)	−1.53	[−2.31, −0.74]	0.0001	88	*P* < 0.00001

## Data Availability

All data compiled or analyzed during this study are included within this article.

## Abbreviations

CNKI: China National Knowledge Infrastructure, https://http://www.cnki.net/
CQVIP: WEIPU information network, https://http://www.cqvip.com
CBM: China Biology Medicine disc, http://http://www.sinomed.ac.cn/
PSQI: Pittsburgh Sleep Quality Index.

## Additional Points

In this study, the number of included literatures and cases was limited, and the methodological quality was relatively low. Only 17 studies described the specific random methods in detail, 6 studies implemented allocation concealment and 5 studies implemented blind evaluation of results, which may affect the accuracy of this study. The PSQI score of the outcome index is subjective, and the effective rate is calculated based on the PSQI score, which reduces the objectivity of the evidence in this study to a certain extent.
